# Isolation of clinically relevant concentrations of bone marrow mesenchymal stem cells without centrifugation

**DOI:** 10.1186/s12967-018-1750-x

**Published:** 2019-01-05

**Authors:** Michael Scarpone, Daniel Kuebler, Andrew Chambers, Carlo Maria De Filippo, Mariangela Amatuzio, Thomas E. Ichim, Amit N. Patel, Eugenio Caradonna

**Affiliations:** 1 Trinity Sports Medicine and Performance Center, Trinity Hospital, Steubenville, OH 43952 USA; 2 Department of Biology, Franciscan University of Steubenville, Steubenville, OH 43952 USA; 3 The Centre of Research and Formation High Technologies “Johannes Paulus II”, Catholic University of Campobasso, Campobasso, Italy; 4 Creative Medical Technologies, Phoenix, AZ USA; 5 Department of Bioengineering, University of Utah, Salt Lake City, USA

**Keywords:** Bone marrow aspirate, Hematopoietic stem cells, Mesenchymal stem cells

## Abstract

**Background:**

This study examined the quality of bone marrow aspirates extracted using a novel, FDA cleared method to optimally target cells from the inner cortical iliac bone surface without the need for centrifugation. This method employs small draws from a single puncture that promote only lateral flow from multiple sites (SSLM method). The study utilized the Marrow Cellutions bone marrow aspiration system (MC system) which is based on the SSLM method and compared the MC system directly to bone marrow concentrates (BMAC) generated by centrifugation of aspirates harvested with a standard aspiration needle.

**Methods:**

Three direct comparisons were conducted evaluating the SSLM draws and BMACs derived from the same patient from contralateral iliac crests. The levels of TNCs/mL, CD34+ cells/mL, CD117+ cells/mL, and CFU-f/mL were compared between the various bone marrow preparations. The cellular content of a series of SSLM draws was also analyzed to determine the total nucleated cell (TNC) count and the concentration of mesenchymal stem/progenitor cells as measured by colony forming unit fibroblasts (CFU-f).

**Results:**

In direct comparisons with BMAC systems, SSLM draws yielded significantly higher CFU-f concentrations and comparable concentrations of CD34+ and CD117+ cells. In addition, the average quantity of TNCs/mL in a series of 30 patients utilizing the SSLM draw was 35.2 × 10^6^ ± 17.1 × 10^6^ and the average number of CFU-f/mL was 2885 ± 1716. There were small but significant correlations between the TNCs/mL and the CFU-fs/mL using the SSLM method as well as between the age of the patient and the CFU-fs/mL.

**Conclusions:**

The MC Device, using the SSLM draw technique, produced concentrations of CFU-fs, CD34+ cells and CD117+ cells that were comparable or greater to BMACs derived from the same patient. Given the rapid speed and simplicity of the MC Device, we believe this novel system possesses significant practical advantages to other currently available centrifugation based systems.

## Background

Bone marrow aspirate (BMA) and concentrates (BMAC) have been used to treat a variety of orthopedic injuries as well as critical limb ischemia and heart failure with positive clinical outcomes being linked to higher numbers of stem/progenitor cells in the bone marrow sample [1–7]. Because of their clinical importance, a variety of techniques exist to increase the yield of stem/progenitor cells in bone marrow aspirate [6, 8]. Unfortunately, attempts to aspirate excess bone marrow (a volume over 1 mL from a single site) will introduce significant peripheral blood that has lower numbers of stem/progenitor cells into the aspiration [9–11]. In addition, given the variety of niches in which stem/progenitor cells reside within the bone and marrow space [12–14], different aspiration techniques may be more or less effective at preferentially extracting cells from different niches.

A common option to increase the concentration of stem/progenitor cells in large volume aspirates is to concentrate the aspirate by density gradient separation using a centrifuge-based system [6, 11]. While BMAC systems can increase the concentration of nucleated cells in the aspirate [11], because they do not distinguish between peripheral blood and marrow components, they may merely increase the level of peripheral blood nucleated cells, which contain very few stem/progenitor cells.

A novel approach to potentially overcome this issue is to utilize the Marrow Cellutions bone marrow aspiration system (MC system) which employs multiple small volume draws (1 mL) from a single puncture that promote lateral flow from multiple sites near the inner cortical bone space in the bone marrow (SSLM method). It is known that this anatomical location contains a high concentration of bone marrow stem/progenitor cells [12–14]. In addition, The aspiration cannula used to employ this method is: (1) closed on the end to limit peripheral blood infiltration from the distal end of the cannula, (2) has side holes to preferentially draw cells from the sides closest to the inner cortical plate verses the center of the iliac bone, and (3) has a mechanical feature to precisely re-position the side aspiration ports within the medullary space.

In this study, we performed three direct comparisons between SSLM derived bone marrow aspirates and BMAC systems using contralateral iliac crest draws from the same patient. In comparison 1, the SSLM method was compared to Harvest BMACs derived from three puncture sites. In comparison 2, the SSLM method was compared to Harvest BMACs derived from a single puncture site. Finally, in comparison 3, the SSLM method was compared to EmCyte BMACs derived from a single puncture site. The levels of TNCs/mL, CFU-fs/mL, CD34+/mL and CD117+/mL were examined in these comparisons to determine differences in cellular components obtained using SSLM and BMAC systems. Finally, we measured the levels of TNCs/mL and CFU-fs/mL harvested by the SSLM method in a series of 30 patients. We found that the MC system produced concentrations of CFU-fs, CD34+ cells and CD117+ cells that were comparable or greater to BMACs derived from the same patient.

## Materials and methods

Three different direct comparisons were performed between the SSLM method (MC system) and different BMAC systems. In all three cases, contralateral iliac crest draws from the same patient were used for comparison. In the first comparison, the levels of TNCs, CFU-fs, and CD34+ cells obtained using the SSLM method (single puncture) were compared to BMACs obtained via three punctures from the same patient’s contralateral hip. In the second comparison, the levels of TNCs, CD34+ cells, and the related hematopoietic progenitor CD117+ cells obtained via the SSLM method (single puncture) were compared to BMACs obtained via a single puncture using the same BMAC system as comparison one. To determine if the specific centrifuge system influenced the results, the third comparison tested an additional centrifugation system. In this case, the levels of TNCs, CFU-fs, and CD34+ cells obtained via the SSLM method (single puncture) were compared to BMACs obtained via a single puncture using a different BMAC system. Finally, the cellular content of a series of SSLM draws was analyzed to determine the total nucleated cell (TNC) count and the concentration of mesenchymal stem/progenitor cells as measured by CFU-fs to see how the levels of CFU-fs using the SSLM method compared to previously published thresholds for CFU-f levels required for clinical effectiveness.

### Bone marrow aspiration and concentration

All bone marrow aspirates (BMA) were taken from the posterior superior iliac spine. A careful review of the manufacturer’s most recent instructions for use, (written instructions and on-line video) prior to aspiration and processing was performed for the Harvest BMAC system, the EmCyte BMAC system and the Marrow Cellution (MC) system which employed the SSLM method. The EmCyte and Harvest BMACs were processed within the procedure room following the manufacturers recommendations. For the SSLM method, aspirates were performed along the insertion trajectory, starting distal to the point of entry into the medullary space, with the device mechanically repositioning the aspiration ports to a new location after each 1 mL of aspiration. BMA was aspirated using a 10 mL syringe using quick sharp pulls of the plunger at each 360 degree turn. For each 1 mL draw, the aspiration ports were moved approximately 3/4 cm out of the body. A total of 8 to 12 mL of bone marrow was harvested. Samples were then analyzed at one of three laboratories using the protocols described below. Lab 1: Franciscan University of Steubenville, Steubenville, OH. Lab 2: BSR Laboratories, Cambridge, MA. Lab 3: The Centre of Research and Formation High Technologies “Johannes Paulus II”, Catholic University of Campobasso. The study was approved by the Franciscan University Institutional Review Board and the Catholic University of Campobasso Institutional Review Board and informed consent was obtained from all participants.

### First centrifuge comparison

Bone marrow aspirate samples were collected during spine surgery from the iliac spine by Clinician A from five patients undergoing elective orthopedic procedures. BMA was aspirated from randomly assigned alternate iliac crests, using either the Harvest BMAC system or the MC system. While Clinician A had significant experience using the Harvest BMAC system, it was this clinician’s first attempt at using the MC system. Aspiration of 20 mL from three separate punctures was performed for the Harvest BMAC sample for centrifugation; a single puncture was performed with the MC system using the technique described above. Laboratory analysis was performed at Lab 2. TNC levels were counted utilizing an automated analyzer (Coulter Ac.T diff2). CD34+ cells concentrations [15] and CFU-f levels [16] were analyzed using methods described previously. All samples were processed within 24 h of collection.

### Second centrifuge comparison

Five sets of bone marrow aspirate samples were collected from a single puncture in patients with end stage critical limb ischemia from each iliac spine by Clinician B. BMA was aspirated from randomly assigned alternate iliac crests, using either the Harvest BMAC system or the MC system. This was Clinician B’s first attempt at using the MC system. The levels of TNCs, CD34+ cells, and CD 117+ cells in the samples were then analyzed at Lab 3 using their previously published methodologies [17]. All samples were processed within 8 h of collection.

### Third centrifuge comparison

Bone marrow aspirate samples were collected from a single puncture from each iliac spine, randomly assigned to alternate iliac crests, using either the EmCyte BMAC system or the MC system in three patients undergoing elective orthopedic procedures. The MC Device was used by Clinician C using the same method described above. Samples were then analyzed in Lab 1 to determine TNC/mL, CD34+ cells/mL, and CFU-f/mL levels.

To determine TNC levels, the BMA was diluted 1:20 in Hank’s Balanced Salt Solution (HBSS) and mixed 1:1 with AO/PI dye (Nexcelom Bioscience). The sample was then counted using a Cellometer Vision CBA Cytometry System (Nexcelom Bioscience) to determine the concentration of live and dead nucleated cells. All samples were counted in duplicate.

To measure CFU-f levels, 5–10 μLs of undiluted bone marrow was plated in 6-well dishes containing 3 mLs of DMEM/F12 media (GIBCO) supplemented with 15% MSC qualified FBS (GIBCO) and an antibiotic/antimycotic mix (Gibco). Cells were cultured under standard conditions, 37 °C, 5% CO_2_. After 48 h, the wells were washed four times with HBSS to remove non-adherent cells. The cells were then cultured for 12 additional days with the media being changed every 3 days. After 14 days total, the colonies were stained with 0.5% crystal violet solution in methanol. Colonies with 100 or more cells were counted as CFU-fs. All samples were processed in duplicate.

The levels of CD34+ cells were determined using the established ISHAGE protocol [18]. Briefly, 50 μLs of marrow was mixed with 40 μLs of cell staining buffer, 5 μLs of PE conjugated CD34+ antibody (Biolegend) and 5 μLs of FITC conjugated CD45+ antibody (Biolegend) and incubated for 20 min at RT. RBC lysis buffer was then added and samples were then run on an Accuri flow cytometry using gates established by the ISHAGE protocol. All samples were processed within 8 h of collection.

### Case series

A series of 30 consecutive patients from the same clinician (Clinician C) underwent marrow aspiration using the MC system for use in elective orthopedic procedures. Clinician C had previous experience using the MC system as described above. Samples were then analyzed in Lab 1 to determine TNC/mL and CFU-f/mL levels using the methods described above. All samples were processed within 8 h of collection.

### Statistics

For the three different comparisons between the MC system and centrifugation systems, TNC, CFU-f, CD34+ and CD117+ levels were compared using paired student t-tests. Prism software was used for all the above statistical analyses. For the case series of 30, the age, TNC counts, and CFU-f counts are reported as average counts ± SD. The data were analyzed for normal distribution using a Shapiro–Wilk normality test. Correlations between TNC counts and CFU-f counts were analyzed using a nonparametric Spearman’s correlation as the TNC counts were not normally distributed. Correlations between patient age and CFU-f counts were analyzed using Pearson’s correlation as both age and CFU-f counts were normally distributed. Linear regression was performed on each of the two sets of data.

## Results

### MC system vs. centrifugation comparison #1

The MC system was compared in five patients with Harvest processed BMACs obtained from the contralateral iliac crest (Table 1). Three separate punctures were made to aspirate the marrow used for the centrifugation process while only one puncture site was used for the MC system. The mean TNC count for the MC system was 31.5 ± 15.5 million/mL vs. 66.1 ± 20.5 million/mL for a traditional needle aspirate (three punctures) followed by processing using the Harvest centrifuge-based system (p = 0.0001). Likewise, the mean CD34+ count for the MC system was 71.2 ± 49.0 × 10^3^/mL vs. 254 ± 122 × 10^3^/mL for the Harvest system (p = 0.0034). Finally, the mean CFU-f count for the MC system was 1583 ± 858/mL vs. 796.6 ± 508.5/mL for the Harvest system (p = 0.0346). For all three measurements, the values were significantly different with the Harvest system having significantly elevated TNC and CD34+ counts and the MC system having significantly higher CFU-f counts.

**Table 1:** MC system vs. Harvest centrifugation system; comparison 1

	Marrow cellution			Harvest system		
	TNCs/mL × 10^6^	CFU-fs/mL	CD34+/mL × 10^3^	TNCs/mL × 10^6^	CFU-fs/mL	CD34+/mL × 10^3^
Patient 1	55.0	2915	157	96.0	1248	435
Patient 2	27.2	1278	66	64.0	1312	308
Patient 3	16.6	552	49	40.3	232	203
Patient 4	20.5	1496	42	57.5	883	209
Patient 5	38.0	1672	42	72.5	308	117
Average	31.5 ± 15.5**	1583 ± 858*	71.2 ± 49.0**	66.1 ± 20.5	797 ± 509	254 ± 122

Levels of TNCs, CFU-fs, and CD34+ cells in MC aspirates (single puncture site) and Harvest bone marrow concentrates (three punctures sites) taken from contralateral iliac crest draws in the same patient. (* p < 0.05; ** p < 0.01)

### MC system vs. centrifugation comparison #2

Harvest processed BMACs and MC system prepared aspirates were also compared in a second group of patients (n = 5) with the aspirations being drawn by a different clinician (Table 2) but in this case only one puncture was made to aspirate the marrow used for BMAC processing. The mean TNC count for the MC system, 24.2 ± 11.6 million/mL, was significantly lower than the value found with the Harvest processed BMAC system, 39.7 ± 17.2 million/mL (p = 0.0454). The mean CD34+ count for the MC system in this comparison was 257 ± 115 × 10^3^/mL vs. 217 ± 143 × 10^3^/mL for the Harvest system, a difference that did not reach statistical significance (p = 0.1378). CFU-f counts were not performed on these samples, however, the level of CD117+ cells was examined in this comparison. The average CD117+ count for the MC system was 272 ± 141 × 10^3^/mL vs. 180 ± 164 × 10^3^/mL for the Harvest system, a difference that did not reach statistical significance (p = 0.0530).

**Table 2:** MC system vs. Harvest centrifugation system; comparison 2

	Marrow cellution			Harvest system		
	TNCs/mL × 10^6^	CD34+/mL × 10^3^	CD117+/mL × 10^3^	TNCs/mL × 10^6^	CD34+/mL × 10^3^	CD117+/mL × 10^3^
Patient 1	23.1	235	348	15.7	91.2	89.7
Patient 2	15.3	135	84.4	28.3	164	76.5
Patient 3	16.4	177	193	44.8	125	89.6
Patient 4	44.1	423	450	55.8	447	463
Patient 5	22.1	315	282	53.7	258	183
Average	24.2 ± 11.6*	257 ± 115	272 ± 141	39.7 ± 17.2	217 ± 143	180 ± 164

Levels of TNCs, CD34+, and CD117+ cells in MC aspirates and Harvest bone marrow concentrates taken from contralateral iliac crest draws in the same patient. (* p < 0.05)

In addition to testing the two systems in this comparison, analysis was also performed on a sample of the traditional needle aspirate before it was processed using the Harvest system. When the traditional needle aspirate prior to centrifugation was compared with the MC system, the TNC counts (12.56 ± 2.6 million/mL vs. 24.2 ± 11.6 million/mL), the CD34+ counts (68.8 ± 30.7 10^3^/mL vs. 257 ± 115 10^3^/mL), and the CD117+ counts (65.0 ± 44.0 10^3^/mL vs. 272 ± 141 10^3^/mL) were all significantly reduced as compared to the MC system. Finally, the CD34+ counts were highly correlated with the CD117+ counts in both the MC system (Pearson’s correlation coefficient = 0.894; p = 0.041), and the Harvest system (Pearson’s correlation coefficient = 0.966; p = 0.007).

### MC system vs. centrifugation comparison #3

In the final comparison, a second BMAC centrifugation system was compared with the MC system. In this case, the MC system was compared in three patients with EmCyte processed BMACs obtained from the contralateral iliac crest (Table 3). The mean TNC count for the MC system was 36.7 ± 17.7 million/mL vs. 60.5 ± 53.8 million/mL for the traditional needle aspirate followed by processing with the EmCyte centrifuge-based system (p = 0.1888). The mean CD34+ count for the MC system was 237 ± 88 × 10^3^/mL vs. 146 ± 33 × 10^3^/mL for the EmCyte system (p = 0.0806). Likewise, the mean CFU-f count for the MC system was 2263 ± 1667/mL vs. 267 ± 76.4/mL for the EmCyte system (p = 0.0437). While the mean values showed considerable differences for all three comparisons between the MC and EmCyte systems, the difference was only significant in the case of the elevated CFU-f levels found in the MC system preparation (p < 0.05).

**Table 3:** MC system vs. EmCyte centrifugation system; comparison 3

	Marrow cellution			EmCyte system		
	TNCs/mL × 10^6^	CFU-fs/mL	CD34+/mL × 10^3^	TNCs/mL × 10^6^	CFU-fs/mL	CD34+/mL × 10^3^
Patient 1	57.2	3610	326	121	350	151
Patient 2	25.9	1560	150	18	250	110
Patient 3	27.1	1620	236	42	200	176
Average	36.7 ± 17.7	2263 ± 1667*	237 ± 88	60.5 ± 53.8	267 ± 76.4	146 ± 33

Levels of TNCs, CFU-fs, and CD34+ cells in MC aspirates and EmCyte bone marrow concentrates taken from contralateral iliac crest draws in the same patient. (* p < 0.05)

### TNC and CFU-f levels in a case series of 30 MC system aspirates

**Fig. 1:**
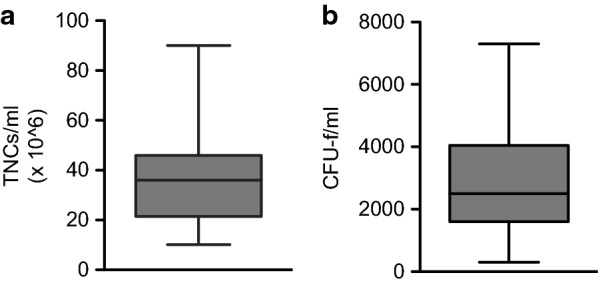
Number of CFU-fs and TNCs in bone marrow aspirates obtained using the MC system (SSLM method). **a** The average number of TNCs/mL was 33.3 × 10^6^ ± 13.9 × 10^6^ when removing the outlier case (90.0 million TNCs/mL). When all cases are included, the average count was 35.2 × 10^6^ ± 17.1 × 10^6^ (n = 30). **b** The average number of CFU-fs/mL was 2885 ± 1716 (n = 30)

A series of 30 bone marrow aspirates harvested using the MC system were analyzed for TNC and CFU-f levels. The TNC counts for these aspirates ranged from 10.1 to 57.2 million TNCs/mL with a single statistical outlier having a 90.0 million TNCs/mL count (Table 4). The mean and standard deviation for the TNC count was 35.2 million TNCs/mL ± 17.13 million (Fig. 1a). The median TNCs/mL value was 36.0 million. While the range of TNC counts for women was shifted lower (10.1–38.2 million) than the range for men (13.6–57.2 million), no statistical difference could be inferred due to the low number of women [4] included in the study. The CFU-f counts for the 30 samples ranged from 300 to 7300 CFU-fs/mL (Table 4). The mean and standard deviation for the CFU-f count were 2885 CFU-fs/mL ± 1717 (Fig. 1b) with a median value of 2500. The average number of CFU-fs per million nucleated cells was 99.8 ± 123.5. There was significant variation in the samples as values ranged from 11.4 to 722.8

**Table 4:** The TNC and CFU-f counts for BMA samples drawn from 30 patients using the MC system (SSLM method)

Nuc cells/mL	CFU’s/mL	Sex	Age	Co morbidities
44.6 × 10^6^	4200	Male	52	HTN, GERD
36.3 × 10^6^	3600	Male	45	Crohns, GERD
21.6 × 10^6^	3200	Male	72	HTN, CAD
15.5 × 10^6^	2000	Male	59	None
46.1 × 10^6^	2500	Male	37	Low IGF-1
26.4 × 10^6^	300	Male	85	RA, diabetes, gout, used Humira
44.2 × 10^6^	2500	Male	57	None
15.6 × 10^6^	1200	Female	65	Hypothyroid
37.4 × 10^6^	6700	Male	22	None
20.9 × 10^6^	2400	Male	69	None
38.2 × 10^6^	2900	Female	76	HTN
17.8 × 10^6^	700	Male	58	Diabetes, obesity
35.7 × 10^6^	4000	Female	34	Psoriatic arthritis
48.5 × 10^6^	2400	Male	46	GERD, smokes
51.0 × 10^6^	5400	Male	44	HTN, GERD, gout, obesity
15.3 × 10^6^	800	Male	56	None
44.6 × 10^6^	4200	Male	65	Ulcers
10.1 × 10^6^	7300	Female	61	None
45.9 × 10^6^	2610	Male	68	OA, gout, high BP, kidney, liver damage
13.6 × 10^6^	780	Male	69	None
24.7 × 10^6^	1830	Male	55	None
31.5 × 10^6^	4670	Male	56	None
42.9 × 10^6^	2000	Male	60	Border line diabetes
48.4 × 10^6^	2390	Male	78	None
23.4 × 10^6^	1110	Male	70	None
57.2 × 10^6^	3610	Male	65	OA, gout, HTN
55.2 × 10^6^	3835	Male	21	None
90.0 × 10^6^	4225	Male	23	None
25.9 × 10^6^	1560	Male	65	OA, HTN, anxiety
27.1 × 10^6^	1620	Male	43	Kidney stones, HTN, testosterone

Between 8 and 10 mLs of bone marrow aspirate was drawn from each of the patients. The gender, age and co-morbidities of each patient are also listed

Of the four individuals who had CFU-f counts below 1000/mL, two had significant co-morbidities (diabetes, obesity, RA, and gout). The other two had no co-morbidities, nor were they outliers based on age as both were within one standard deviation of mean age of the population tested. Of the seven individuals with CFU-f counts above 4000/mL, four had no co-morbidities. Of the three that exhibited co-morbidities, one had ulcers, another hypertension and GERD while the most complex case had hypertension, GERD, gout and obesity (Table 4). Thirteen of the 30 individuals included in the study had no co-morbidities. These individuals had an average CFU-f count of 3118/mL ± 2115 while those with co- morbidities had an average count that was not significantly different, 2705/mL ± 1381.

### Age and TNC correlations with CFU-f levels in MC system aspirates

**Fig. 2:**
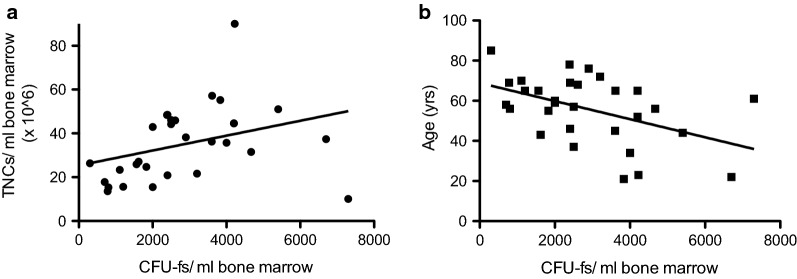
**a** Correlation between CFU-fs/mL and total nucleated cells/mL in bone marrow aspirates harvested using the SSLM method. Because the data were not normally distributed, the Spearman’s rank-order correlation test was used. The Spearman coefficient was 0.4848, indicating a positive correlation between the two variables that was significant (p = 0.0062). **b** Correlation between CFU-fs/mL and age in bone marrow aspirates harvested using the SSLM method. Because the data were normally distributed, the Pearson’s correlation test was used. The Pearson correlation coefficient was − 0.4689, indicating a negative correlation between the two variables that was significant (p = 0.009)

We examined whether a correlation existed between the TNC counts and the CFU-f counts in the MC system aspirates. Figure 2a shows the CFU-f count for each sample plotted against the TNC count. The Spearman’s correlation coefficient for the data is 0.48837 indicating a weak positive correlation between the two values that was significant (p = 0.0062). If the two-outlier points are removed (patients 7 and 28), an even stronger positive association is seen as the Spearman’s correlation coefficient is 0.6263 (p = 0.0004). Given the reduction in regenerative ability that occurs with age, we examined whether a decrease in CFU-f counts in bone marrow aspirate was associated with the age of the patient. Figure 2b shows the CFU-f count for each sample plotted against the age of the patient. The data show a weak negative correlation between age and CFU-f number with a Pearson’s correlation coefficient of − 0.4689 (p = 0.009), indicating that older patients tend toward lower CFU-f counts.

## Discussion

BMA and BMACs, which contain a complex mix of nucleated cells, platelets and growth factors, are known to aid in the healing of a variety of orthopedic injuries and to promote angiogenesis in ischemic tissue [2, 19–22]. While BMA and BMACs show significant promise in the clinic, it is important to validate individual bone marrow preparations given the numerous methods for aspirating and concentrating bone marrow currently in use [11] as well as the different locations of stem cells within the marrow space [13, 14]. This is particularly important given that aspirates with higher levels of CFU-f cells and/or CD34+ cells have been shown in a variety of studies to correlate with improved patient outcomes [1–4, 21].

Given the association between higher levels of stem/progenitor cells and positive patient outcomes, verifying that BMAs and BMACs contain sufficient numbers of these cells is of clinical relevance. In this study, we found that the MC system which employs the SSLM method, produces ~ 10 mL aspirate with high concentrations of CFU-fs and/or CD34+ cells. Most traditional needle aspirates are sourced from a cannula with an open distal end without a mechanical means to precisely reposition the cannula. These traditional needle techniques normally produce aspirates that contain between 200 and 300 CFU-f/mL [11]. In the three direct comparisons with three different clinicians and two different centrifuge systems, the MC system had significantly higher amounts of CFU-fs and tended toward similar or higher numbers of CD34+ and CD117+ cells as compared to centrifugation systems (Tables 1, 2, 3). The only exception was the significantly higher levels of CD34+ cells in the first comparison where the BMAC draw was performed using smaller aspirations at three different insertion sites, as opposed to a single large aspiration at only one insertion site as performed in the other two comparisons. Despite the elevated CD34+ cells in this BMAC, the MC system aspirate still had significantly higher CFU-f levels. Interestingly, the smaller volume aspirations done at the three different insertion sites for the BMAC draw seemed to preferentially increase the number of CD34+ cells without a corresponding increase in CFU-f levels. Previous studies have shown while the CFU-fs are predominately found in the CD34+ population, only a small fraction of CD34+ cells are capable of forming CFU-fs so the levels may not always correlate [23].

There is increasing evidence that different MSC populations are found in unique niches within the bone. MSC populations have been identified within the cortical bone space and trabecular bone cavities [12, 24], as well as within endosteal and perivascular niches within the marrow space [13, 25]. It is possible that different aspiration techniques can preferentially target these subpopulations. The BMAC aspirates were done using an aspiration needle with an open-ended cannula that has five side ports but that draws preferentially through the largest distal open end that is oriented toward the center of the bone space. In contrast, the MC system draws only from the sides and not the center as the distal opening is closed. Given these differences, the endosteal population near the inner cortical bone space [13], located adjacent to the side ports in the MC system, may be preferentially targeted with the SSLM method. As a result, while multiple small incisions/draws using a traditional needle may increase the CD34+ count, this technique may target a cell population from the inner marrow space that is not as rich in CFU-f forming cells. Our data supports the hypothesis that a different cell population richer in MSCs may reside along the endosteal niche near the cortical surface of the bone, a population that can be preferentially targeted based upon the aspiration method.

High concentrations of CFU-fs were found utilizing the MC system by two different clinicians using the analysis of two different independent laboratories. In the case series, the MC system produced 10 mL aspirates that had an average of 2885 CFU-f/mL. The total number of CFU-fs found in the MC system aspirates, roughly 28,000, represents a level of progenitor/stem cells that is in line with concentrations from other studies that have shown therapeutic success in reducing disc pain [26], treating hip osteonecrosis [27] and ameliorating cartilage defects [1]. In addition, the number of CFU-fs/mL found here in the case series was greater or similar than those reported previously for centrifuge-based final products [11, 21].

Overall, the data presented here challenges the rationale for aspirating large volumes of bone marrow and then volume reducing through centrifugation. While centrifugation systems allow for higher TNC levels as seen here, they did not in general produce BMACs with higher CFU-f, CD34+ or CD117+ concentrations than the MC system. The CFU-f/mL yields in the BMACs tested here are at the low end of the range as compared with previous BMAC studies that found CFU-f/mL yields in the 500–3000 range [11, 16, 21, 26, 28]. The high end of this range is similar to what was found here with the MC system case series. Based upon the CFU-f, CD34+ and CD117+ levels, the comparison data suggest that higher concentrations of HSCs and MSCs are found following the SSLM method [29].

There are several likely explanations for the high levels of stem/progenitor cells harvested with the SSLM method. First, the MC system automatically repositions the aspiration cannula allowing it to mimic multiple puncture sites each with small 1 mL aspirations. Previous studies have shown that large volume aspirates (more than 2 mL) from a single site using traditional needles tend to be infiltrated by significant amounts of peripheral blood, which contains very few MSCs and HSCs and leads to lower CFU-f and CD34+ cell counts [9, 10]. This is particularly important in diabetic, atherosclerotic and elderly patients in which the amount of MSC and HSC is significantly reduced [30–32]. Secondly, the MC system aspirates from side ports, thereby (1) pulling in fluid from across a greater geography of the marrow space and (2) targeting the endosteal niche closest to the inner cortical plate, a region known to be rich in stem cells [13, 25]. While enzymatic digestion is necessary to fully harvest MSCs from this site, aspiration of marrow from this region appears to yield more MSCs than marrow harvested from traditional needle systems. Finally, the inefficiencies of the centrifugation process can leave significant numbers of stem/progenitor cells behind in the discarded portion of the processed marrow. Studies have shown that many MSCs are present as aggregates in bone marrow aspirates, aggregates that can be removed by the filtering and centrifugation steps associated with BMAC preps [33, 34].

In addition, very small embryonic-like stem cells (VSELs), which are found in BM and can contribute to tissue regeneration, are lost during common centrifugation protocols due to their high nucleo-cytoplasmic ratio [35, 36]. Given the variety of cells in the bone marrow that can aid in tissue regeneration (HSCs, MSCs, VSELs) [33, 37, 38] and the fact that significant amounts of these cells can be lost in the centrifugation process, the ability to avoid centrifugation can be a significant benefit in preparing a robust biologic.

In order to better characterize the aspirates generated by the MC system, we examined the correlation between TNC and CFU-f counts obtained from MC system aspirates. It was found that the two values did show a small but highly significant correlation with higher TNC values associated with higher CFU-f counts. Previous studies using BMAC have found a similar correlation [28]. In addition, we found a small but significant correlation between age and CFU-f counts using this process, with aspirates from older individuals correlating with lower CFU-f yields. These results are in general agreement with previous research [39].

While the data presented here demonstrates that following the SSLM method produces an aspirate that contains high CFU-f and CD34+ levels, there are limitations associated with the study. First, while suitable for a pilot study, the sample size for each of the three comparison studies was small. Second, the different methodologies at the three labs were not standardized. However, the fact that CFU-f values were comparable between the two labs that tested CFU-fs suggests that the differences in protocols did not substantially affect the outcome of the study. Despite this, future studies should include a larger sample of side-by-side comparisons with standard laboratory protocols in place. Given the possibility that different aspiration techniques can preferentially target different bone marrow niches, further studies investigating the geographical location of different cell types within the marrow space and the influence of different aspiration protocols on selecting these cells is warranted. Finally, given that higher numbers of MSCs as measured by CFU-fs have been associated with positive treatment outcomes, future studies should include follow-up on patients treated with these aspirates.

## Conclusions

This study demonstrates that bone marrow samples containing relatively high CFU-fs/mL and CD34+/mL can be obtained without the need for centrifugation using the MC system. In side by side comparisons with BMACs procured from the same patients using the contralateral iliac crest, the level of CFU-fs/mL was significantly higher in the MC system. In addition, MC system aspirates had similar numbers of CD34+ and CD117+ cells as compared to centrifugation systems. Finally, the CFU-f/mL levels obtained using the MC system were positively correlated with the TNC count and negatively correlated with the age of the patient. As a result, knowing the TNC count and age of the patient can provide some information regarding the potential quality of the bone marrow aspirate obtained using the MC system.
